# Tension Stimulation of Tenocytes in Aligned Hyaluronic Acid/Platelet-Rich Plasma-Polycaprolactone Core-Sheath Nanofiber Membrane Scaffold for Tendon Tissue Engineering

**DOI:** 10.3390/ijms222011215

**Published:** 2021-10-18

**Authors:** Chih-Hao Chen, Dai-Ling Li, Andy Deng-Chi Chuang, Banendu Sunder Dash, Jyh-Ping Chen

**Affiliations:** 1 Department of Plastic and Reconstructive Surgery, Chang Gung Memorial Hospital at Keelung, Keelung 20401, Taiwan; chchen5027@gmail.com (C.-H.C.); andy.d.chuang@gmail.com (A.D.-C.C.); 2 Department of Plastic and Reconstructive Surgery and Craniofacial Research Center, Chang Gung Memorial Hospital at Linkou, Collage of Medicine, Chang Gung University, Taoyuan 33305, Taiwan; 3 Department of Chemical and Materials Engineering, Chang Gung University, Taoyuan 33302, Taiwan; jennifer86130@hotmail.com (D.-L.L.); banendusunder@gmail.com (B.S.D.); 4 Research Center for Food and Cosmetic Safety, College of Human Ecology, Chang Gung University of Science and Technology, Taoyuan 33305, Taiwan; 5 Department of Materials Engineering, Ming Chi University of Technology, New Taipei City 24301, Taiwan

**Keywords:** aligned nanofibers, tendon tissue engineering, electrospinning, platelet-rich plasma, hyaluronic acid, bioreactor

## Abstract

To recreate the in vivo niche for tendon tissue engineering in vitro, the characteristics of tendon tissue underlines the use of biochemical and biophysical cues during tenocyte culture. Herein, we prepare core-sheath nanofibers with polycaprolactone (PCL) sheath for mechanical support and hyaluronic acid (HA)/platelet-rich plasma (PRP) core for growth factor delivery. Three types of core-sheath nanofiber membrane scaffolds (CSNMS), consisting of random HA-PCL nanofibers (Random), random HA/PRP-PCL nanofibers (Random+) or aligned HA/PRP-PCL (Align^+^) nanofibers, were used to study response of rabbit tenocytes to biochemical (PRP) and biophysical (fiber alignment) stimulation. The core-sheath structures as well as other pertinent properties of CSNMS have been characterized, with Align^+^ showing the best mechanical properties. The unidirectional growth of tenocytes, as induced by aligned fiber topography, was confirmed from cell morphology and cytoskeleton expression. The combined effects of PRP and fiber alignment in Align^+^ CSNMS lead to enhanced cell proliferation rates, as well as upregulated gene expression and marker protein synthesis. Another biophysical cue on tenocytes was introduced by dynamic culture of tenocyte-seeded Align^+^ in a bioreactor with cyclic tension stimulation. Augmented by this biophysical beacon from mechanical loading, dynamic cell culture could shorten the time for tendon maturation in vitro, with improved cell proliferation rates and tenogenic phenotype maintenance, compared to static culture. Therefore, we successfully demonstrate how combined use of biochemical/topographical cues as well as mechanical stimulation could ameliorate cellular response of tenocytes in CSNMS, which can provide a functional in vitro environmental niche for tendon tissue engineering.

## 1. Introduction

Tendinopathy is a common condition negatively affecting the life quality of laborers, athletes and physically active individuals, accounting for up to 30% of musculoskeletal consultation in general practice [1]. With the function to transfer forces generated by muscles to the bone to induce joint motion, tendons are constantly exposed to high tensile forces, rendering them vulnerable to microtraumas, strains, and ruptures as a result of acute or chronic trauma. Coupled with the poor intrinsic repairing ability of tendons from their hypovascularity and hypocellularity, treatment for tendinopathy often requires more aggressive approaches [2]. While adjunct treatment options such as growth factor and gene therapy are available, current clinically available options for tendon ruptures are mostly confined to tendon prosthesis, allografts, mosaicplasty and autografts [3]. Despite advances made in prosthetic materials, poor biodegradation and extensive formation of scar and fibrotic tissue from poor biocompatibility may limit the widespread adaptation of tendon prosthesis [4]. Allografts, while possessing better biocompatibility than artificial prostheses, carry the risk of immunogenicity and disease transmission. Although tissue processing methods such as fresh-freezing, cryo-preservation, ethylene oxide treatment, and gamma irradiation can decrease the risks, detrimental effects on biomechanical properties of the grafts cannot be ruled out [5]. To address these pitfalls, tissue engineering approaches by seeding tendon-derived cells on scaffolds with unique characteristics have yielded promising results, which can provide optimal growth environment while facilitating cellular proliferation and extracellular matrix (ECM) deposition [6,7].

The electrospun nanofiber membrane is a popular choice for use as a scaffold in tissue engineering [8]. Desirable attributes can be introduced into nanofibers by changing their structure or by imbuing them with drugs or signaling molecules for controlled focal delivery [9]. Changes could be made to the orientation of nanofibers as well. The electrospun nanofibers are usually deposited randomly on a static collector. However, by using a rapidly rotating collector, nanofibers can be collected in a coordinated manner to fabricate a membrane scaffold consisting of aligned nanofibers. A membrane scaffold composed of aligned nanofibers is deemed suitable to provide seeded cells with topographical cue to guide directional cellular growth, as well as enhanced cell proliferation and differentiation to generate functional ligamentous and tendinous tissues [10,11]. This anisotropic structure could also fortify the tensile strength of the scaffold while responding to a directional stimulus, such as uni-axial mechanical loading. Hence, aligned fiber topography is expected to provide a proper biophysical cue in aiding cell elongation along the direction of the aligned fibers with improved cellular function and proliferation rate [12]. Furthermore, as tendons are composed of closely packed anisotropic collagen fibers, it is also desirable to use a membrane scaffold with architectural features emulating tendon morphology, to maintain the physiological functions of tenocytes during in vitro cell culture. Through unique characteristics of aligned nanofibers, favorable tenocyte cell culture environment could be realized to promote cell growth, induce tenogenic phenotypes and upregulate tendon-specific genes [13].

The platelet-rich plasma (PRP) is a blood plasma fraction with platelet-rich cellular components [14]. It is enriched with various growth factors such as insulin-like growth factor-1 (IGF-1), platelet-derived growth factor-BB (PDGF-BB) and basic fibroblast growth factor (bFGF). Those growth factors in PRP plays key roles during normal tendon development and tendon wound healing [15], increases tenocyte proliferation and ECM formation [16], and is beneficial for the regeneration of tendons [17]. In addition, pathological tendons may be benefited from growth factors in PRP, by promoting cellular proliferation and support angiogenesis [18]. For this reason, PRP administration is gaining popularity as a treatment for tendon injuries. Considering the use of PRP, the growth factors and mitogens found in PRP can be incorporated into electrospun nanofibers to increase its bioactivity. A study directly added lyophilized powder of PRP, after a freeze-thaw cycle to lyse platelets, to a polymer-containing spinning solution prepared from organic solvents [19]. A limitation of this blend electrospinning technique is that growth factors from PRP may lose their bioactivity and even denature in the presence of organic solvents [20]. To avoid direct electrospinning of polymer/PRP mixtures, electrospun nanofibers prepared from a spinning solution containing polymers only could be coated with PRP by impregnation and freeze-dried [21]. Nonetheless, this method did not offer sustained release of proteins from adsorbed PRP. Alternatively, emulsion electrospinning could be employed by embedding PRP into nanofibers with an emulsion spinning solution to achieve sustained release of growth factors [22]. Although emulsion electrospinning may provide protection of growth factors during preparation, the high temperature generated during the electrospinning process is detrimental for their bioactivity [23]. Considering those limitations, co-axial electrospinning is deemed suitable to fabricating PRP-loaded core-sheath nanofibers [24]. By avoiding direct contact between the core and sheath solutions, bioactive proteins in PRP could be incorporated within the core by using a weak acid core solution, while protected from harmful organic solvents in the sheath solution, which can also eliminate the initial burst release of growth factors [25]. Considering culture of tendon-derived cells, it is also desirable to combine hyaluronic acid (HA) in the core with PRP. The HA plays an important role in maintaining good quality tendon ECM, as well as creating an ideal environment for cell nutrition and lubrication in tendon [26]. It is also noted that tendon ECM is composed predominately of collagen fibers and three key components, HA (HA), glycosaminglycans (GAGs) and proteoglycans (PGs), with GAGs and PGs both containing HA. Furthermore, HA also increases the production of collagen due to the stimulation of tenocytes, as well as stimulates the conversion of collagen III in fibrotic tissue of unhealthy tendon into collagen I in healthy tendon [27].

In tendons, the mechanotransduction is regulated by tenocyte cytoskeleton such as primary cilia and gap junctions [28]. Therefore, mechanical loading during tenocyte culture could be a useful biophysical tool to maintain the phenotype of tenocytes. A previous study indicated that mechanical loading could maintain the phenotype and innate elongated cell morphology of tenocytes to increase expression of tendon-specific genes as well as synthesis of marker proteins [29]. Although such characteristics are indicative of an enhanced intrinsic reparative process during tendon healing, prolonged and excessive stretching were reported to induce inflammatory response [30]. Overloading tendon cells can lead to elevated levels of matrix metalloproteinases that deteriorate ECM integrity [31]. For mechanical loading during in vitro culture of tenocytes, moderate uni-axial stretching was shown to elevate marker protein production and tenogenic differentiation without eliciting inflammatory responses [32]. As biophysical cues from topography and mechanical loading both play key roles in modulating the in vitro microenvironment during tenocyte culture, combined use of engineered scaffold fabrication techniques and bioreactor systems is desirable [33]. These features may be incorporated into a single protocol for tenogenic phenotype maintenance in vitro using aligned core-sheath nanofibers and commercially available bioreactors for dynamic cell culture.

In this study, we hypothesize the combination of biochemical and biophysical signaling during tenocyte culture can recreate native tendon structure in vitro by maintaining the tenogenic phenotype of tenocytes. For this purpose, we prepare three types of core-sheath nanofiber membrane scaffold (CSNMS), random nanofibers with HA core and polycaprolactone (PCL) sheath (Random), random nanofibers with HA/PRP core and PCL sheath (Random^+^), and aligned nanofibers with HA/PRP core and PCL sheath (Align^+^). With PRP as a biochemical cue and fiber alignment as a topographical modulator, we characterize the physicochemical characteristics of the scaffolds as well as their cellular response with rabbit tenocytes. The best CSNMS from static cell culture was further chosen for cell culture in a bioreactor subject to cyclic tension stimulation, for achieving the best tenogenic phenotype maintenance under niche provided both biochemically (PRP) and biophysically (fiber alignment and mechanical stimulation).

## 2. Results and Discussion

### 2.1. Physicochemical Properties of Core-Sheath Nanofiber Membrane Scaffolds (CSNMS)

**Figure 1:**
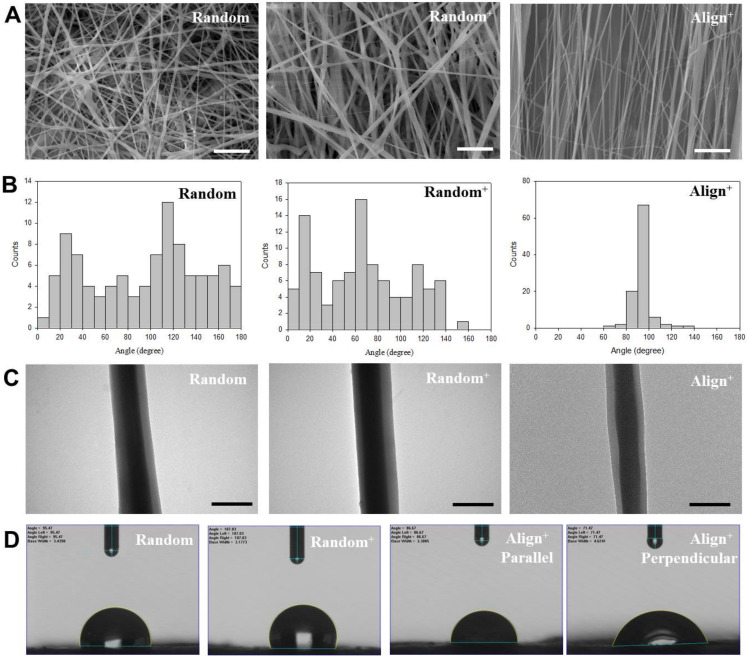
The scanning electron microscope (SEM) images (**A**) (bar = 10 μm), fiber angle distribution (**B**), transmission electron microscope (TEM) images (**C**) (bar = 500 nm), and optical micrographs of water droplets for contact angle measurements (**D**) of core-sheath nanofiber membrane scaffold (CSNMS). The water contact angles were determined from direction both parallel and perpendicular to longitudinal axis of nanofibers in Align^+^ CSNMS.

The scanning electron microscopy (SEM) images of CSNMS reveals smooth fiber surface morphology from nanofibers with uniform diameter and no bead formation (Figure 1A). The fiber diameters are 410 ± 96 nm, 483 ± 116 nm and 362 ± 138 nm for Random, Random^+^ and Align^+^ CSNMS, respectively, using the ImageJ software for analysis. Although no significant difference was noticed in fiber size, reduced mean fiber diameter for Align^+^ coincide with faster solvent evaporation rate, generated from forced convection air flow around a rotating collector to collect aligned nanofibers. The incorporation of PRP also leads to increased mean fiber diameter as shown between Random and Random^+^. Nonetheless, by controlling fiber diameter within a fixed range for different CSNMS, the impact arising from unique features associated with CSNMS (i.e., PRP and fiber alignment) on tenocyte phenotypes could be elucidated. Considering fiber orientation, substantial differences in fiber angle distribution was noted for scaffolds composed of random or aligned nanofibers (Figure 1B), which is also well demonstrated from SEM images in Figure 1A. The nanofibers of Align^+^ demonstrate low variation of orientation from the vertical position (90°) with 87% fibers showing fiber angles within 80 to 100°. In contrast, Random and Random^+^ show substantial alignment deviation with wide distribution of fiber angles from 0 to 180° (Figure 1B). Undoubtedly, deviation from alignment is expected for Align^+^, as collecting nanofibers with a rapidly rotating collector can lead to repulsion of deposited fibers from incomplete charge release [34]. The biophysical cue provided by fiber alignment will induce seeded tenocytes to produce ECM resembling the reparative process after tendon injury, where aligned nanofiber topography can coincide with the nanoscale extracellular structure of healthy tendon for tenocytes to maintain their phenotype [35].

The transmission electron microscopy (TEM) images clearly reveal details of the core-sheath nanofiber structure for all CSNMS (Figure 1C). The core width (core diameter) and the sheath width (fiber diameter − core diameter) was estimated by ImageJ and reported in Table 1. Though there is no statistical significance, a smaller mean core width of Random, compared with Random^+^ or Align^+^, may be due to absence of PRP. Table 1 also shows the porosity of CSNMS, where significantly lower value was found for Align^+^ than the other two groups. This could arise as aligned nanofibers will pack more densely within the membrane than their random counterparts, which also significantly increases the density of Align^+^ (Table 1) [36]. It is therefore conceivable that well-aligned nanofibers will lead to a denser membrane structure with lower porosity for Align^+^. This feature may potentially provide scaffolds with improved mechanical strength, a trait that is highly coveted in the context of tendon tissue engineering. 

The water contact angles of CSNMS reveal surface tension of water droplet on the membrane, with a larger contact angle value reflecting reduced hydrophilicity (Figure 1D). Contact angles from Random and Random^+^ revealed no significant difference, indicating composition difference within the core did not influence surface wettability of the scaffold (Table 1). The apparent hydrophobicity of these scaffolds is consistent with the presence of hydrophobic polymer PCL in the nanofiber sheath, though the geometric potential theory is also a reasonable explanation for the perceived hydrophobicity of CSNMS [37,38]. The water contact angles of Align^+^ were determined from two different directions during measurements, parallel or perpendicular to the longitudinal axis of fibers. It is expected that the anisotropy of aligned fibers will lead to direction-dependent change of water contact angles, in contrast to isotropic water wetting for randomly oriented nanofibers. As shown in Table 1, while measurements taken parallel to the nanofiber axis show no significant difference in contact angle, the value measured from direction perpendicular to fiber orientation is significantly lower than both Random and Random^+^. This lower contact angle is due to preferential spreading of the water droplet along the axis of the nanofiber, with no barriers to motion of the contact line. Since the geometric potential theory cannot be used in this case, the apparent decrease of contact angles indicates higher hydrophilicity for Align^+^, when observed perpendicularly to the nanofiber axis than parallelly [38,39].

**Figure 2:**
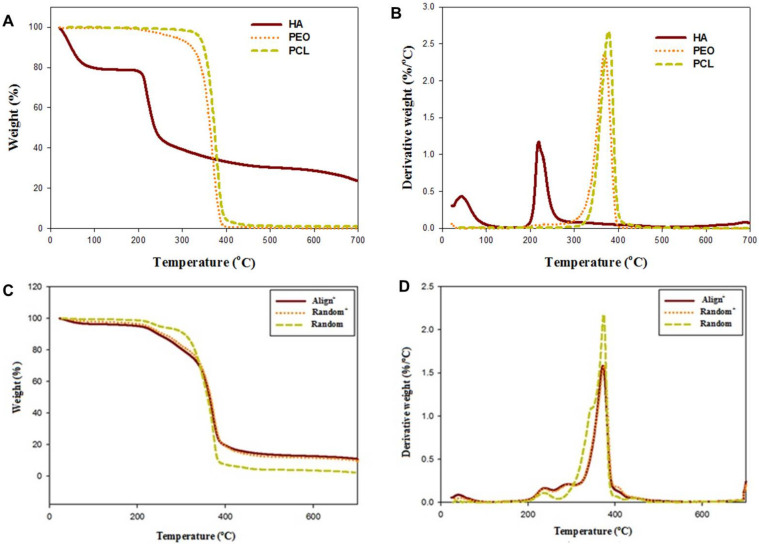
The thermogravimetric analysis (TGA) (**A**,**C**) and derivative thermograms (**B**,**D**) of component materials (**A**,**B**) and Random, Random^+^ and Align^+^ core-sheath nanofiber membrane scaffold (CSNMS) (**C**,**D**). HA: hyaluronic acid, PEO: polyethylene oxide, PCL: polycaprolactone.

The thermal stability of component materials in CSNMS is first analyzed by thermogravimetric analysis (TGA) and shown in Figure 2A. The derivative thermograms show peak decomposition temperatures at 378.1 and 369.0 °C for PCL and polyethylene oxide (PEO) found in the sheath compartment (Figure 2B). For the core, HA demonstrates early weight loss from 60 °C due to evaporation of associated water. The thermal breakdown of the natural polymer HA occurs earlier than the synthetic polymers (PEO and PCL), with a peak temperature at 231.9 °C. The natural polymers HA shows some residual weight after burning in nitrogen to 700 °C (23.6%), in contrast to ~0% for synthetic polymers PCL and PEO [40]. For CSNMS, the TGA shows nearly identical decomposition curves for Align^+^ and Random^+^, implying similar chemical composition between them (Figure 2C). This was expected, as the only difference between them is fiber orientation. A peak at ~236 °C for all CSNMS reflects the presence of HA, while a peak extending from 371 to 375 °C reflects the combined effect of PCL and PEO (Figure 2D). The residual mass at 700 °C for Random (2.3%) also shows a distinctive feature, which is noticeably lower than Aligned^+^ and Random^+^ (~10.1%), due to the absence of PRP in the core of nanofibers.

**Figure 3:**
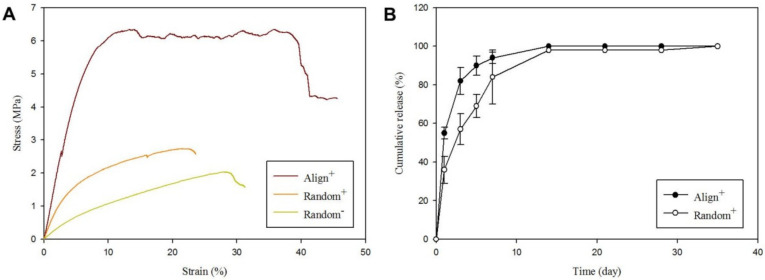
(**A**) The mechanical tensile testing of Random, Random^+^ and Align^+^ core-sheath nanofiber membrane scaffold (CSNMS). (**B**) The in vitro release of proteins from Random^+^ and Align^+^ CSNMS after incubating in PBS at 37 °C. The cumulative release percentage is calculated based on the amount of proteins released on day 35, which is taken as 100%.

For tensile mechanical properties of different CSNMS, the stress-strain curves indicate Align^+^ has the best mechanical properties (Figure 3A). The Young’s modulus of Align^+^ is 76.02 MPa, which is 4.1 times and 8.5 times that of Random^+^ and Random (Table 2). The orientation of fibers thus dramatically affects the mechanical properties of CSNMS, with aligned fibers provides a stiffer scaffold with a higher Young’s modulus, compared to random fibers [41]. The ultimate stress and ultimate strain are also significantly higher for Align^+^ than Random^+^ and Random (Table 2). Overall, these findings are consistent with previous findings where higher tensile strength was found for membrane scaffolds composed of aligned nanofibers [10,11,42]. Nonetheless, crystallinity difference between aligned and random fibers may also influence the observed mechanical properties. The degree of crystallinity of aligned PCL nanofiber membranes was found to be higher than that of random PCL nanofiber membranes [43]. This may increase tensile strength in addition to the effect of fiber alignment.

Protein release rates of PRP-containing CSNMS (Align^+^ and Random^+^) were measured with the Bio-Rad Protein Assay (Figure 3B). With the amount of protein released at the end of experiment (day 35) taken as 100%, the cumulative release profiles of proteins were assessed at different time points. Compared to Align^+^, Random^+^ demonstrates lower protein release rate during the first 2 weeks, which might arise from the difference in sheath width (Table 1), where thinner sheath in Align^+^ is expected to promote faster protein diffusion rate from the core reservoir. However, the higher specific surface area associated with Align*^+^* may also contribute to this phenomenon. We have measured the specific surface areas of CSNMS, which are 17.9 and 28.5 m^2^/g for Random*^+^* and Align*^+^*. The smaller fiber size of Align*^+^* provides larger specific surface area for faster protein release when compared with Random*^+^*. However, the difference in crystallinity of PCL shell between the membranes can also influence the release rate. After two weeks, the cumulative amount of protein released plateaued, where both scaffolds have essentially released all proteins in PRP from the nanofibers. This release rate is ideal as growth factors in PRP are paramount in the early phases of tendon injury by promoting cell migration, recruiting tendon-derived cells, enhancing cell viability, and inducing DNA synthesis [44]. By releasing the core contents early while still maintaining its structure with the PCL sheath, the CSNMS can provide mechanical support for growth of attached tenocytes. As described before, we incorporate PRP within the core by preparing the PRP-containing core solution with a weak acid (formic acid) to avoid direct contact with the harmful organic solvents in the sheath solution. To confirm whether formic acid will denature the released growth factors in PRP after released, we measured the release profiles of PDGF-BB from Random*^+^* and Align*^+^* by using enzyme-linked immunosorbent assay (ELISA). As shown in Figure S1, the percentage of growth factor released approaches 100% after two weeks, after normalizing with the growth factor content in the CSNMS (determined from ELISA) before electrospinning.

### 2.2. In Vitro Cell Culture

**Figure 4:**
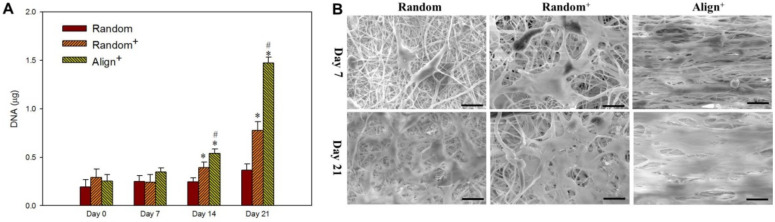
The cell proliferation rates from DNA assays (**A**) and the scanning electron microscopy (SEM) images (**B**) (bar = 20 μm) of tenocytes cultured on Random, Random^+^ and Align^+^ core-sheath nanofiber membrane scaffold (CSNMS). * *p* < 0.05 compared with Random, ^#^ *p* < 0.05 compared with Random^+^.

As methylene chloride (MC) and *N*,*N*′-dimethyl formamide (DMF) mixture was used to prepare the shell solution while formic acid was used to prepare the PRP-containing core solution, the effects from these harmful and toxic solvents on tenocytes should be aware and examined. To confirm that the growth factors were active and working on the cells, we extracted different CSNMS with 2 mL cell culture medium for 3 days. The extract was used for culture tenocytes on tissue culture polystyrene (TCPS) and the viable cell number was determined from MTS assay at different times. As shown in Figure S2, the extract from Random^+^ and Align^+^ could substantially accelerate the extent of tenocyte proliferation in vitro, as reported for pristine PRP [15,45]. The proliferation of tenocytes in CSNMS was determined from DNA contents. There is no difference in cell attachment rate on day 0; however, distinctive feature shown from the rate of DNA increase underlines proliferation of tenocytes in CSNMS is different (Figure 4A). The cells in Align^+^ shows the highest cell proliferation rate, whereas Random^+^ is also significantly higher than Random. Therefore, PRP or aligned fiber topography will elicit faster cell proliferation rate, as mediated by respective biochemical or biophysical cue. As the difference between Random and Random^+^ is only PRP, the higher cell proliferation rate observed in Figure 4A for Random^+^ may be assigned to PRP, which provides a biochemical cue. Similarly, as the difference between Random^+^ and Align^+^ is only fiber alignment, difference in cell response between them could be provided by the biophysical cue from fiber alignment. By observing cell-seeded scaffolds under SEM, the difference in abundance of well-spread cells supports cell growth rate from DNA assays (Figure 4B). Furthermore, tenocytes show random cellular structure, and the secreted ECM showed an omnidirectional web-like extension for Random^-^ and Random^+^. In contrast, tenocytes in Align^+^ shows elongated shape with the long axis parallel to the nanofiber orientation, as well as unidirectional deposition of ECM. The perceived directional secretion of ECM is likely associated with the apparent directional topography of the nanofibers, which can guide cell growth along the axial direction of nanofibers. Our findings thus mirror with those reported in the literature, where geometric potential theory has been used to explain this phenomenon [46]. By culturing cells in Align^+^ with topographic cues from parallel nanofibers, a relatively uni-directional cell growth pattern is observed, which would be important for tenocytes to maintain their physiological functions in vitro [47]. As Align^+^ is composed of highly organized nanofibers, similar to collagen fibers alignment in native tendon, it is expected to offer mechanical stability for cell growth, while improving the structural organization of the newly formed tissue-like construct with re-differentiated tenocyte phenotype.

**Figure 5:**
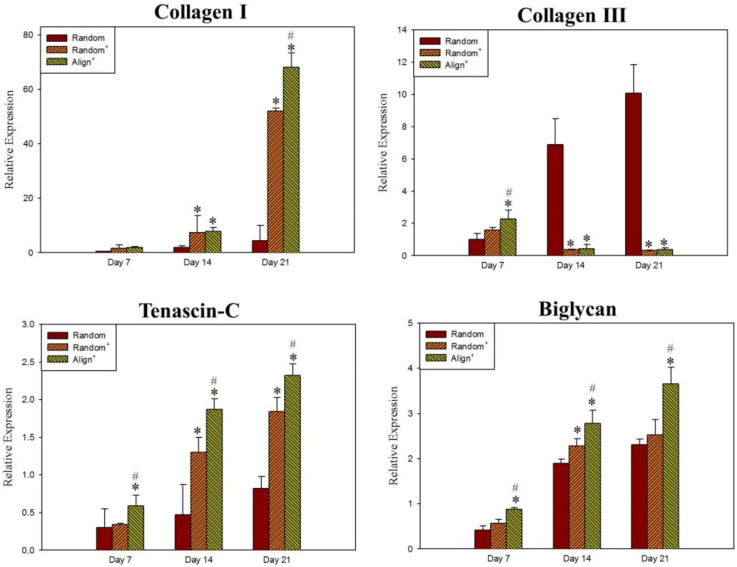
The relative mRNA expression of type I collagen (collagen I), type III collagen (collagen III), tenascin-C and biglycan by tenocytes after cultured in Random, Random^+^ and Align^+^ core-sheath nanofiber membrane scaffold (CSNMS) for 7, 14 and 21 days. * *p* < 0.05 compared with Random, ^#^ *p* < 0.05 compared with Random^+^.

Normal tendon consists mostly of type I collagen (collagen I). During tendon healing, both collagen I and type III collagen (collagen III) are synthesized by tenocytes, with collagen III being produced predominantly during the earlier phase of tendon healing [48]. The collagen III is converted to denser and stronger collagen I as scar tissues mature, where greater mechanical strength is required. From gene expression analyzed by quantitative real-time polymerase chain reaction (qRT-PCR), collagen III is readily expressed in all CSNMS during the early phase on day 7 (Figure 5). By day 14, Random^+^ and Align^+^, but not Random, show significant down-regulation of collagen III gene expression, when shift of collagen III to collagen I occurs during tendon maturation. Concomitantly, the collagen I gene expression is up-regulated on day 14 for Random^+^ and Align^+^, over that of Align (Figure 5). The shift of gene expression from collagen III to collagen I is likely due to the presence of growth factors in proteins released from Random^+^ and Align^+^ [49]. This pattern of gene expression continues to day 21, with collagen I gene expression in Align^+^ further surpassing that of Random^+^, suggesting the contribution of nanofiber topographical cues towards tenocyte maturation [50]. Together, with significant upregulation of collagen III gene expression early on day 7, the gene expression patterns of different types of collagen underscores the importance of using Align^+^ to maintain tenocyte phenotype in vitro. The glycoprotein tenascin-C and the proteoglycan biglycan are both integral parts during tenocyte proliferation and tendon repair. Tenascin-C, important in the establishment and maintenance of fibrocartilaginous regions of tendons, is found to be significantly upregulated in Random^+^ and Align^+^, compared to Random [51]. Furthermore, Aligned^+^ displays significantly higher tenascin-C mRNA expression level than Random^+^, exemplifying the most efficient maintenance of tenocyte phenotype with this scaffold. Similar results could be found for biglycan, a proteoglycan associated with thick collagen fibrillogenesis and organization [52].

**Figure 6:**
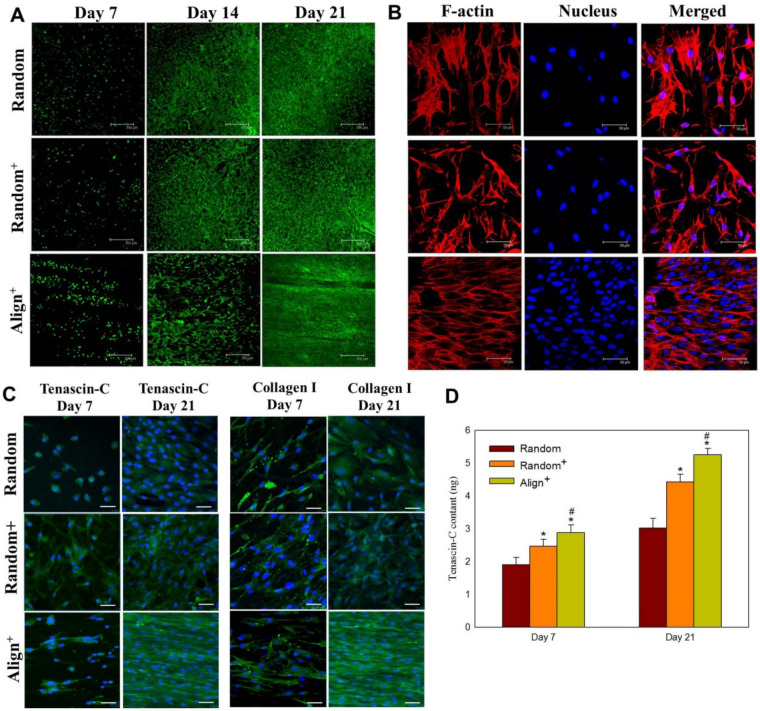
The Live/Dead staining (**A**) (bar = 300 μm), cytoskeleton arrangement on day 21 (**B**) (bar = 50 μm), immunofluorescence staining of tenascin-C and collagen I (**C**) (bar = 50 μm) and tenascin-C protein synthesis (**D**) after culture tenocytes in Random, Random^+^ and Align^+^ core-sheath nanofiber membrane scaffold (CSNMS). * *p* < 0.05 compared with Random, ^#^ *p* < 0.05 compared with Random^+^.

The confocal microscopy images from Live/Dead staining show time-dependent increase of green fluorescence signal from live cells, whereas negligible dead cells with red fluorescence was found for tenocytes in all CSNMS, supporting the high cell viability during cell growth (Figure 6A). The difference in number of viable cells between groups is consistent with cell proliferation rates measured from DNA contents in Figure 4A. The directional cell growth could be also inferred from distribution of the fluorescence signal, where only tenocytes on Aligned^+^ show cell growth in one direction, echoing results from SEM observation. The mechanism of nanofiber alignment to guide cell growth was further elucidated by staining F-actin in cell cytoskeleton with phalloidin-tetramethylrhodamine B isothiocyanate (phalloidin-TRITC), and counterstained with 4,6-diamidine-2-phenylindole dihydrochloride (DAPI) for cell nucleus. As shown in Figure 6B, the cytoskeletal structures of tenocytes growing on Align^+^ indicate cellular extension parallel to fiber alignment. Additionally, the cell nuclei in Align^+^ conform to the stretching of cells, appearing fusiform with cell alignment in the direction of cytoskeletal distribution. Cells growing on Random and Random^+^ retain relatively spherical-shaped nuclei, while cytoskeletal extension remains omnidirectional. These findings provide further evidence for the impact of nanofiber alignment on direction of cellular growth. As topographical-induced signaling is essential for physiological function maintenance in vitro, we could foresee possible reinstating of lost phenotype with the physiologically relevant elongated cell morphology for tenocytes grown in Align^+^ [47]. The cytoskeletal structures of tenocytes on Align^+^ show cellular extension parallel to fiber alignment with cell nuclei conforming to the direction of cytoskeletal distribution. In contrast, tenocytes grown on Random and Random^+^ retain relatively spherical-shaped nuclei with omnidirectional cytoskeletal extension. The topographical feature offered by aligned nanofiber in Align^+^ is therefore an attractive reprogramming mediator during tenocyte culture, which may overcome limited efficiency from biological supplements alone [53].

The synthesis of tendon maker proteins was confirmed from confocal microscopy with immunofluorescence (IF) staining (Figure 6C). For both tenascin-C and collagen I, the protein expression levels are in the order Align^+^ > Random^+^ > Align. The directional distribution of collagen I in Align^+^ is identical to that shown from cytoskeleton distribution in Figure 6B. This confirms the direct effect of collagen I on actin organization and cellular elongation, which forms aligned collagen fibrils as the basic building blocks of tendons [54]. Tenascin-C, another key fibroelastic ECM protein found in tendon, show similar pattern to that of collagen I [55,56]. With the presence of PRP in Random^+^ and Align^+^, transforming growth factor (TGF) and epidermal growth factor (EGF) can both stimulate cellular production of tenascin-C, while TGF and insulin-like growth factor (IGF) contribute to the synthesis of collagen I [49]. Undoubtedly, the pronounced synthesis of tendon marker proteins supports the high expression of tendon-specific genes (Figure 5), which endorse of the combined use of biochemical (PRP) and biophysical (fiber alignment) cues for the benefits of matrix protein synthesis of tenocytes. However, to confirm the difference in protein production levels observed from IF staining, we used enzyme-linked immunosorbent assay (ELISA) to directly quantify the amount of tenascin-C produced by tenocytes. As shown in Figure 6D, we could verify time-lapsed and scaffold-dependent production of tenascin-C from tenocytes in different CSNMS.

Tenocytes cultured in vitro tend to dedifferentiate, after which the dedifferentiated tenocytes may suffer from drift of tenocyte phenotype with altered morphology and decreased expression of tenogenic markers [57]. This lack of healthy tendon niche demands the use of biochemical and biophysical cues during in vitro tenocyte culture to drive re-differentiation of tenocytes to rescue them from their lost tenocyte phenotype [58]. From the above results, we have successfully used proteins (growth factors) released from PRP-loaded Random^+^ and Align^+^ as a supplement during cell culture for re-differentiation of dedifferentiated tenocytes in vitro, as shown from increased expression of marker genes [59]. Conversely, we also successfully manipulate tenocyte shape by attaching cells to anisotropic fibers in Align^+^, where a topographical biophysical cue further push cells into an elongated shape, which is beneficial for maintenance of tenocyte phenotype [60]. All these explored elements are expected to promote the formation of a tissue-engineered tendon in vitro, for possible application as a tissue replacement in vivo. Such a combined approach for tendon tissue engineering clearly demonstrates benefits from cooperative effects from dual factors in Align^+^ CSNMS, which may be augmented further by another biophysical cue from dynamic cell culture. 

### 2.3. Dynamic Cell Culture

**Figure 7:**
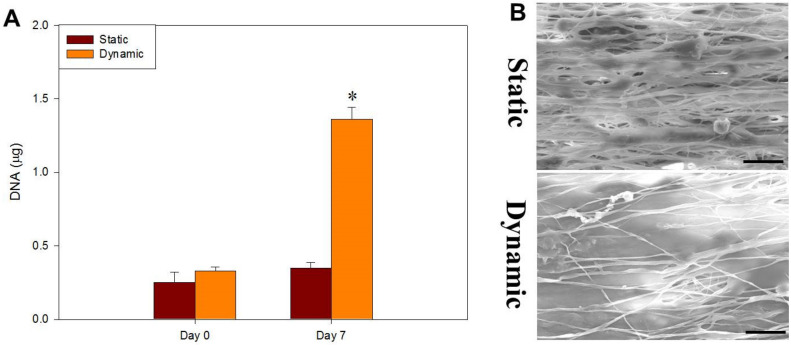
The proliferation (**A**) and scanning electron microscopy images (**B**) (bar = 20 μm) of tenocytes after static or dynamic culture for 7 days in Aligned^+^ core-sheath nanofiber membrane scaffold (CSNMS). * *p* < 0.05 compared with static.

Tendons are constantly exposed to mechanical stimuli from surrounding ECM or neighboring cells. This mechanotransduction, which helps cells in tendons to adapt to continuous dynamic stress from the microenvironment, can lead to beneficial intracellular molecular processes after transforming such a biophysical cue into biological responses [61]. With the high ultimate strain of CSNMS, the best scaffold found from static culture (i.e., Align^+^) was used for dynamic cell culture, by exposing the cell-seeded scaffold to cyclic tension stimulation in a bioreactor for 7 days, by operating at 3 h stimulation time per day, 6% strain rate and 1 Hz frequency. As shown in Figure 7A, the proliferation of tenocytes is enhanced with dynamic culture, with increase of DNA content to 3.9 folds compared with static culture. This difference is also revealed from SEM observation, where a dramatic increase of cell density was found for tenocytes cultured dynamically in a bioreactor, compared to those in a cell culture plate statically (Figure 7B). The ECM secreted by tenocytes also markedly enhanced when under repeated mechanical stress to mimic conditions found during tenocyte growth, where mechanical loading can induce favorable phenotyping [62].

**Figure 8:**
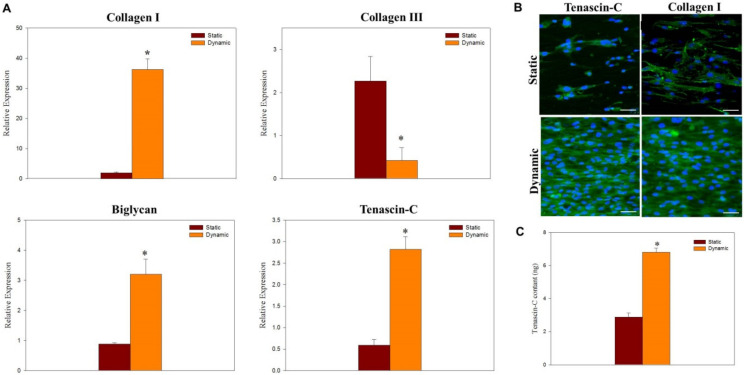
The relative mRNA expression (**A**), immunofluorescence staining of tenascin-C and collagen I (**B**) (bar = 50μm) and tenascin-C protein synthesis (**C**) after static or dynamic culture for 7 days in Aligned^+^ core-sheath nanofiber membrane scaffold (CSNMS). * *p* < 0.05 compared with static.

For gene expression under repeated tension stimulation, upregulation of collagen I, biglycan, and tenascin-C and downregulation of collagen III genes is found from qRT-PCR, indicating mechanotransduction from tension stimulation in dynamic culture can induce faster tendon maturation (Figure 8A). Other than beneficial for growth of tenocytes, controlled mechanical loading during dynamic culture may also increase the expression of tendon-specific genes via anabolic changes [13]. From confocal microscopy examination, the alignment of tenocytes is preserved or even improved during dynamic culture, when uniaxial tension stimulation was used in the direction of fiber alignment (Figure 8B). A much higher synthesis rate of marker proteins tenascin-C or collagen I is also evident from immunofluorescence (IF) staining (Figure 8B). Finally, the analysis of tenascin-C from ELISA assays directly supports the trend observed from IF staining (Figure 8C). We thus conclude tenocytes show higher proliferation rates and improved tenogenic phenotype maintenance when cultured in a bioreactor with cyclic tension stimulation. It is perceivable that dynamic culture may expedite tendon maturation with concerted regulation of relevant genes during the maturation process and increased cellular proliferation. Overall, by combining mechanical stimulation with the 3D cell culture environment in Align^+^, the best maintenance of phenotype of tenocytes could be achieved. Therefore, exposing tenocytes to biophysical cues in addition to the presence of biochemical cues may improve the outcomes of tendon tissue engineering in vitro. 

## 3. Materials and Methods

### 3.1. Materials

Polycaprolactone (PCL, molecular weight = 80,000 Da), polyethylene oxide (PEO, molecular weight = 2,000,000 Da), Dulbecco’s modified Eagle’s medium (DMEM) high glucose, phalloidin-tetramethylrhodamine B isothiocyanate (phalloidin-TRITC) for F-actin staining and Hoechst 33,258 (bis-benzimide) for DNA quantification were purchased from Sigma-Aldrich (St. Louis, MO, USA). Hyaluronic acid (HA, mean molecular weight = 1.3 × 10^6^ Da) was purchased from Shandong Freda Biochem Co. (Jinan, China). CellTiter 96^®^ AQueous One Solution Cell Proliferation Assay was acquired from Promega Corporation (Madison, WI, USA) and 4,6-diamidine-2-phenylindole dihydrochloride (DAPI) for nuclear staining was purchased from KPL (SeraCare, Milford, MA, USA). Bio-Rad protein assay kit was acquired from Bio-Rad Laboratories, Inc. (Hercules, CA, USA). Rabbit tenascin-C ELISA kit was obtained from BlueGene Biotech (Shanghai, China). Cytiva HyClone™ fetal bovine serum (FBS) was acquired from Thermo Fisher Scientific (Waltham, MA, USA).

### 3.2. Preparation of Platelet-Rich Plasma (PRP)

The preparation of PRP from New Zealand white rabbits (National Laboratory Animal Breeding and Research Center, Taipei, Taiwan) followed similar protocols described before with procedures approved by the Institutional Animal Care and Use Committee of Chang Gung University (IACUC approval No.: CGU107-272, date of approval: 19 March 2019) [63]. In short, whole blood was withdrawn from the central ear artery of a rabbit into a 60 mL syringe containing 5 mL heparin as anticoagulant. The whole blood was centrifuged at 1000× *g* for 15 min. The bottom layer containing red blood cells was discarded while the buffy coat layer containing platelets and plasma was aspirated and further centrifuged at 2000× *g* for 15 min. The precipitated platelet with part of the plasma layer was collected, followed by freeze drying to prepare PRP in powder form for storage. To control the quality of PRP, the platelet number in PRP has been checked by a hematology analyzer, to be ~10 times that of whole blood.

### 3.3. Preparation of Core-Sheath Nanofiber Membrane Scaffold (CSNMS) 

Two different core solutions were prepared. For HA-PCL CSNMS, the core solution was 1.75% (*w*/*w*) HA and 0.5% (*w*/*w*) PEO prepared in formic acid. For HA/PRP-PCL CSNMS, the core solution was 1.75% (*w*/*w*) HA, 0.5% PEO (*w*/*w*) and 5.25% (*w*/*w*) PRP prepared in formic acid. The sheath solution was 8% (*w*/*v*) PCL in methylene chloride (MC) and *N,N’*-dimethyl formamide (DMF) mixture (MC:DMF = 4:1). The scaffold was prepared by co-axial electrospinning with a high-voltage power supply (Glassman; High Bridge, NJ, USA) at 20 kV. Two syringe pumps (KD Scientific, Holliston, MA, USA) were used to deliver core and sheath solutions separately to a co-axial spinneret at 1 mL/h. To collect nanofibers, a collector placed 10 cm from the needle tip was used. For random nanofibers without PRP (Random) or with PRP (Random^+^), the nanofibers were collected with a grounded static collector covered with aluminum foil. For aligned nanofibers with PRP (Align^+^), a grounded rotational drum covered with aluminum foil was used at 2500 rpm rotation rate [64].

### 3.4. Characterization of Core-Sheath Nanofiber Membrane Scaffold (CSNMS)

A CSNMS (0.5 cm × 0.5 cm) was fixed to an aluminum platform with carbon tape and gold-coated at 20 mA for 30 s. The nanofibers were examined with a scanning electron microscope (SEM) (Hitachi S-3000N, Tokyo, Japan) at 15 kV. The fiber diameter was estimated from 100 nanofibers chosen randomly from 5 SEM images (20 fibers each) and analyzed using the ImageJ software (NIH, Bethesda, MD, USA). The fiber alignment was based on the distribution of fiber angles within the same 100 nanofibers. The fiber angle, ranging from 0 to 180°, was obtained from fiber orientation relative to vertical direction (taken as 90°). To evaluate core-sheath morphology of nanofibers, electrospun nanofibers were collected on a copper grid fixed to a collector for 3 to 5 min, followed by examining the copper grid under a transmission electron microscope (TEM) (Hitachi H-7500, Tokyo, Japan). Twenty images (one nanofiber per image) were analyzed to estimate the core width (core diameter) and the sheath width (fiber diameter − core diameter). The porosity was estimated using a specific gravity bottle based on Archimedes’ principle [65]. The density was calculated by dividing the weight of a 3 cm diameter disk-shaped CSNMS with its volume, after measuring membrane thickness with a digital micrometer.

The scaffolds were cut into 0.5 cm × 0.5 cm pieces with 8~10 mg weight and characterized with thermogravimetric analysis (TGA) using a TGA 2050 analyzer (TA Instruments, New Castle, DE, USA). The analysis was from 25 °C to 700 °C at a heating rate of 10 °C/min under nitrogen atmosphere. For contact angles measurement, the membranes were cut into 1.0 cm × 1.0 cm and fixed to specimen holders in an FTA-125 contact angle/surface tension instrument (First Ten Angstroms, Portsmouth, VA, USA). The water contact angle was measured by taking images in 3 s from a drop of distilled water at room temperature. For Random and Random^+^, the measurements were taken randomly, while for Align^+^ they are from directions both parallel and perpendicular to the longitudinal axis of the aligned fiber.

The mechanical tensile testing of CSNMS was carried out uni-axially with a universal tensile testing machine (Tinius Olsen H1KT, Horsham, PA, USA) with a 5 cm × 1 cm rectangle specimen. By vertically mounting with two mechanical grippers to hold both ends, the specimen was left 3 cm gauge length for mechanical loading. A load-deformation curve was obtained using a 10 N load cell at 5 min/min elongation rate. The Young’s modulus, ultimate stress and ultimate strain were obtained from the stress-strain curve. The specific surface area of CSNMS was determined by ASAP 2020 Plus (Micromeritics, Norcross, GA, USA).

For nanofibers containing PRP (Random^+^ and Align^+^), the amount of protein released was used to represent the release of all growth factors. A CSNMS was cut into 1.5 cm disks (~200 mg). The samples were immersed in 1 mL of phosphate buffered saline (PBS) solution (pH 7.4) and incubated at 37 °C. At predetermined times, the PBS was removed and replenished with fresh PBS for up to 5 weeks. The protein concentration in the removed PBS was determined with a Bio-Rad Protein Assay Kit at 562 nm with an ELISA reader. A standard curve was constructed from bovine serum albumin to convert the solution absorbance into protein concentration. The cumulative weight of protein released form CSNMS was determined by adding the total amount of protein released up to a certain time. The cumulative release percentage is calculated by dividing the cumulative released weight at a certain time point by the cumulative release weight on day 35.

### 3.5. In Vitro Cell Culture

#### 3.5.1. Tenocyte Isolation

The protocols to isolate tenocytes were approved by the Institutional Animal Care and Use Committee of Chang Gung University (IACUC approval no.: CGU107-272, date of approval: 19 March 2019). The hind-paws of adult New Zealand white rabbits were removed post-mortem and the extensor digitorum tendons were carefully excised from their tendon sheaths under sterile condition. After complete excision of tendons from the hind-paws, the tendons were placed in a 20% (*w*/*v*) penicillin–streptomycin solution and cut into 0.1 cm long pieces. The pieces were transferred to a T-75 flask filled with cell culture medium (80% DMEM high glucose supplemented with 20% FBS, 1% penicillin-streptomycin, and 3.7 g/L sodium bicarbonate). The flask was incubated at 37 °C under 5% CO_2_ for 14 days, during which tenocytes will migrate from the tendon fragments and attach to inner surface of flask. For expansion of tenocytes, spent medium was removed from the T-75 flask and the flask was washed with 10 mL PBS. The cells were detached from the flask by adding 3 mL of 0.05% trypsin/EDTA and incubated for 3 min, followed by adding 1 mL cell culture medium to stop the reaction. The detached cells were collected in a 15 mL centrifuge tube and centrifuged at 1000× *g* for 5 min. After plating cells in a new T-75 flask, the cells were cultured with fresh cell culture medium with medium changes every three days. Cells from the second and third passages were used for the studies.

#### 3.5.2. Cell Proliferation

The Random, Random^+^ and Align^+^ CSNMS were cut into discs of 1.5 cm diameter and sterilized with ultraviolet (UV) light in an UV box at 100 μJ/cm^2^ for 4 h. The sterilized samples were placed in a 24-well cell culture plate and each membrane was fixed to the bottom of a well using a home-made Teflon O-ring of the same diameter as the well diameter. A 200 μL cell suspension containing 1 × 10^4^ tenocytes was introduced to each scaffold and incubated at 37 °C for 4 h for cell attachment. The disc with attached cells was transferred to a new well and 2 mL of cell culture medium was added to each well for incubation in a CO_2_ incubator at 37 °C, with medium change every 3 days. To determine cell proliferation, the DNA content in each well was quantified on day 0, 7, 14, and 21 by immersing the cell-seeded scaffold in a digestion solution containing 55 mM sodium citrate, 150 mM sodium chloride, 5 mM L-cysteine hydrochloride, 5 mM EDTA and 1 mg papain. After digestion at 60 °C for 24 h, the solution was centrifuged at 1500× *g* for 5 min and DNA content in supernatant was determined by staining with Hoechst 33,258 (bis-Benzimide). An ELISA reader was used to quantify the amount of DNA at 365 nm/458 nm excitation/emission wavelength from a standard curve constructed with calf thymus DNA.

#### 3.5.3. Microscopy Observation

On day 7 and 21, the tenocyte-seeded discs were fixed with 2.5% glutaraldehyde for 2 h, dehydrated through a graded series of ethanol, and dried overnight with hexamethyldisilazane. The dehydrated specimens were mounted on aluminum stubs, fixed with carbon tapes, and sputter-coated with gold at 20 mA for 30 s, after which they were observed under a SEM (S-3000N, Hitachi, Tokyo, Japan) at 10 kV.

A Live/Dead Cell Double Staining Kit (Sigma-Aldrich, St. Louis, MO, USA) was used for simultaneous fluorescence staining of viable and dead cells on day 7, 14, and 21. The staining solution was prepared by mixing 1 mL of PBS buffer solution with 1 μL of calcein-AM solution and 0.5 μL of propidium iodide solution. The resulting mixture was added to each scaffold and incubated for 15 min. After washing with PBS, the cell-seeded scaffold was examined under a confocal laser scanning microscope (Zeiss LSM 510 Meta, Oberkochen, Germany) at excitation 494 nm/emission 517 nm for live cells (green) and excitation 528 nm/emission 617 nm for dead cells (red). For cytoskeletal arrangement, tenocytes were cultured for 21 days, and cell/scaffold was fixed with 4% paraformaldehyde for 30 min and treated with 0.1% Triton X-100. The specimen was washed 3 times with PBS and stained with 20 μL/mL phalloidin-TRITC for 30 min for F-actin. After washing again with PBS, 0.1 μL/mL DAPI was used for counterstaining the nucleus for 10 min. The sample was observed under a laser scanning confocal microscope at excitation 540 nm/emission 545 nm for F-actin and at excitation 340 nm/emission 488 nm for nucleus.

For immunofluorescence (IF) staining of collagen I and tenascin-C, cell-seeded membranes were cultured for 7 and 21 days and fixed with 4% paraformaldehyde for 30 min. After washing the scaffolds with PBST (PBS containing 0.1% Tween 20) for 10 min, non-specific binding was blocked with Hyblock blocking buffer for 1 min, and further washed with PBST for 10 min. The mouse anti-rabbit collagen I or mouse anti-rabbit tenascin-C primary antibody (Abcam, Cambridge, UK)) was added and incubated at 37 °C for 1 h. After washing again with PBST for 10 min, fluorescein isothiocyanate (FITC)-conjugated goat anti-mouse IgG secondary antibody (Jacksons ImmunoResearch Laboratories, Inc., West Grove, PA, USA) was added for binding with the primary antibody at 37 °C for 2 h. The discs were washed again with PBST and counterstained with DAPI for observation under a laser scanning confocal microscope at excitation 490 nm/emission 525 nm for FITC and excitation 340 nm/emission 488 nm for DAPI.

#### 3.5.4. Quantitative Real-Time Polymerase Chain Reaction (qRT-PCR)

The RNA extraction and cDNA synthesis were performed on day 7, 14, and 21 by adding 1 mL of TRIzol^®^ reagent to each specimen and homogenizing the mixture with a homogenizer. The samples were centrifuged at 12,000× *g* for 15 min and supernatant transferred to a new micro-centrifuge tube. Equal volume of 70% ethanol and DEPC-water was added to the micro-centrifuge tube and mixed well. The resulting mixture was used for RNA isolation using standard protocols from a Total RNA isolation kit (GeneDireX, Taipei, Taiwan). The procured messenger RNA (mRNA) was quantified with a micro-spectrophotometer and diluted with DEPC-water. The solution was heated to 55~60 °C for 30 min for complete dissolution of the RNA. Reverse transcription of mRNA into cDNA was performed with SuperScript III reverse transcriptase (Invitrogen, Carlsbad, CA, USA). For quantitative real-time polymerase chain reaction (qRT-PCR), house-keeping gene glyceraldehyde-3-phosphate dehydrogenase (GADPH) was used as internal control. Gene expression of type I collagen (collagen I), type III collagen (collagen III), biglycan, and tenascin-C were measured for each specimen with the primer sequence shown in Table 3. A SYBR™ Green PCR Master Mix and primers (equal parts of forward and reverse primers) (Table 2) for each gene was added. The qRT-PCR analyses were carried out with a RT-PCR system (MiniOpticon Real-Time PCR System, Bio-Rad Laboratories, Inc., Hercules, CA, USA), using the 2^−ΔΔCt^ relative quantification method. An annealing temperature of 53.4 °C was used for GADPH, collagen I and collagen III, while 63.9 °C was used for biglycan and tenascin-C. A total of 50 cycles were performed for each specimen.

### 3.6. Dynamic Cell Culture

For dynamic culture, a bioreactor (ElectroForce^®^ BioDynamic^®^ 5100 Test Instruments, Bose, Eden Prairie, MN, USA) was used to mimic the stretch and relaxation condition experienced by tendons in vivo. The Align^+^ CSNMS was first cut into strips measuring 0.8 cm x 5 cm and sterilized with UV light. The sterilized strip was horizontally mounted in a chamber of the bioreactor, using two tensile grips with 1 cm clearance at both ends, with nanofiber alignment parallel to the force exerted. Each scaffold was seeded with 1 × 10^4^ cells and incubated for 4 h. After cell seeding, the chamber was filled with 250 mL of cell culture medium for dynamic cell culture in a CO_2_ incubator at 37 °C. A tension loading at 1 Hz and 6% strain was applied for 3 h each day. After cultured for 7 days, the cell-seeded scaffolds were subject to DNA quantification, SEM observation, qRT-PCR, cytoskeletal arrangement and IF staining analysis using the same methods described before for static culture.

### 3.7. Statistical Analysis

The data are presented as mean ± standard deviation with statistical analyses performed by SPSS (version 25, IBM SPSS Inc., Chicago, IL, USA). A one-way analysis of variance (ANOVA) was performed for comparisons between groups, while post-hoc analysis with the least significant difference (LSD) test was used with a p value less than 0.05 considered statistically significant. 

## 4. Conclusions

In our work, tenocytes were exposed to a combination of stimuli, including a biochemical stimulus provided by growth factors in PRP, a topographical cue presented by aligned nanofibers, and mechanical induction by applying uni-axial tension stimulation. We first successfully combined biochemical and biophysical cues within a single CSNMS through controlled release of proteins in PRP from the core of aligned core-sheath nanofibers. A CSNMS consisting of aligned nanofibers showed improved mechanical properties with better cellular response. By providing topographical cues from fiber orientation and biochemical signals from PRP, the Align^+^ CSNMS provided the best niche to enhance cell proliferation as well as maintain tenocyte phenotype in vitro. Furthermore, by mimicking the physiological environment of a tendon, mechanical loading provided in a bioreactor during dynamic cell culture affected cell behavior by augmenting the biological and mechanical advantages. Judging from cell proliferation as well as expression of marker genes and proteins, mechanical stimulation can drastically enhance tendon maturation within Align^+^. Overall, through unique design of CSNMS, we successfully employed PRP as a biochemical tool and fiber alignment and mechanical loading as biophysical tools for tenogenic phenotype maintenance in vitro. By performing dynamic culture with Align^+^ in a bioreactor, which simultaneously offered a biochemical cue with PRP as well as a topographical cue from fiber alignment, this study provided multifactorial modality for the development of tendon tissue engineering.

## Figures and Tables

**Table 1:** Properties of different core-sheath nanofiber membrane scaffold (CSNMS).

Properties	Random	Random^+^	Align^+^
Core width (nm)	164 ± 50	242 ± 61	241 ± 107
Sheath width (nm)	228 ± 98	286 ± 106	163 ± 134
BET surface area (m^2^/g)	22.2	17.9	28.5
Porosity (%)	83.1 ± 5.6	86.8 ± 1.8	69.4 ± 3.6 *^,#^
Density (g/cm^3^)	0.28 ± 0.01	0.27 ± 0.02	0.35 ± 0.01 *^,#^
Water contact angle (degree)	94.4 ± 5.1	96.7 ± 6.1	85.7 ± 8.9 (parallel) 73.1 ± 3.0 *^,#^ (perpendicular)

* *p* < 0.05 compared with Random, ^#^ *p* < 0.05 compared with Random^+^.

**Table 2:** The mechanical properties for Random^-^, Random^+^ and Align^+^ core-sheath nanofiber membrane scaffold (CSNMS) from tensile mechanical testing.

CSNMS	Ultimate Stress (MPa)	Ultimate Strain (%)	Young’s Modulus (MPa)
**Random**	2.21 ± 0.99	31.3 ± 1.4	8.90 ± 0.88
**Random^+^**	1.61 ± 0.51	23.63 ± 1.1 *	18.64 ± 4.08 *
**Align^+^**	6.62 ± 0.21 *^,^^#^	45.65 ± 1.0 *^,^^#^	76.02 ± 9.21 *^,^^#^

* *p* < 0.05 compared with Random, ^#^ *p* < 0.05 compared with Random^+^.

**Table 3:** The primer sequence used for quantitative real-time polymerase chain reaction (qRT-PCR) analysis.

Gene		Sequence (5′ → 3′)	Size (Base Pairs)
**GADPH**	Forward	GACATCAAGAAGGTGGTGAAGC	22
	Reverse	CTTCACAAAGTGGTCATTGAGG	22
**Collagen I**	Forward	GCATGTCTGGTTAGGAGAAACC	22
	Reverse	ATGTATGCAATGCTGTTCTTGC	21
**Collagen III**	Forward	AAGCCCCAGCAGAAAATTC	19
	Reverse	TGGTGGAACAGCAAAAATCA	20
**Biglycan**	Forward	AGATCTGCCAGAGACCCTGA	20
	Reverse	ACCCTGGACAGCTTGTTGTT	20
**Tenascin-C**	Forward	CTCTGCACATAGTGAAAAACAATACC	27
	Reverse	TCAAGGCAGTGGTGTCTGTGA	21

## Data Availability

The data presented in this study are available on request from the corresponding author.

## Supplementary Materials

The following are available online at https://www.mdpi.com/article/10.3390/ijms222011215/s1.
