# Supplementary material for: Tension Stimulation of Tenocytes in Aligned Hyaluronic Acid/Platelet-Rich Plasma-Polycaprolactone Core-Sheath Nanofiber Membrane Scaffold for Tendon Tissue Engineering
Source: Int J Mol Sci. 2021 Oct 18;22(20):11215. doi: 10.3390/ijms222011215 (PMC8537129; doi:10.3390/ijms222011215)
Supplement: Supplementary file 1 [file ijms-22-11215-s001.zip › ijms-1395900-supplementary.pdf]

Supplementary Material

Tension Stimulation of Tenocytes in Aligned Hyaluronic Acid/Platelet-Rich Plasma-Polycaprolactone Core-Sheath Nanofiber Membrane Scaffold for Tendon Tissue Engineering

Release of Growth Factor

For nanofibers containing PRP (Random$^+$ and Align$^+$), the release of platelet-derived growth factor-BB (PDGF-BB), an important growth factor in PRP, was determined. A core-sheath nanofiber membrane scaffold (CSNMS) was cut into 1.5-cm disks and immersed in 1 mL of phosphate buffered saline (PBS) solution (pH 7.4) at 37 °C. At predetermined time, the PBS was removed and replenished with fresh PBS for up to 14 days. The amount of released PDGF-BB was quantified using enzyme-linked immunosorbent assay (ELISA) with a PDGF-BB Human ELISA kit from Thermo Fisher Scientific. The cumulative percentage of PDGF-BB released form CSNMS was calculated by normalizing with pristine growth factor content in the CSNMS, determined before electrospinning with ELISA.

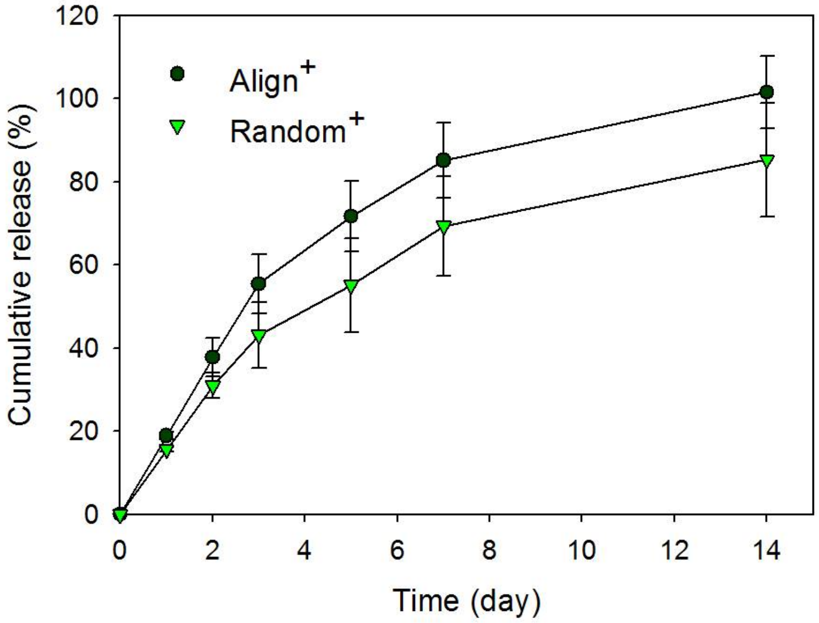

Figure S1. The in vitro release profile of platelet-derived growth factor-BB (PDGF-BB) from Random$^+$ or Align$^+$ core-sheath nanofiber membrane scaffold (CSNMS) in PBS at 37 °C. The amount of released PDGF-BB is quantified with an enzyme-linked immunosorbent assay (ELISA) kit and normalized with pristine PRP used for preparing the CSNMS.

Tenocyte Culture with Extracts from Core-Sheath Nanofiber Membrane (CSNMS)

The Random, Random$^+$ or Align$^+$ CSNMS was extracted separately with 2 mL cell culture medium for 3 days. The extract was used for culture 1 x 10^4 tenocytes/well in a 24-well tissue culture polystyrene (TCPS) cell culture plate. The viable cell number was determined from MTS assay at different time points using the CellTiter 96® AQueous One Solution Cell Proliferation Assay kit from Progema.

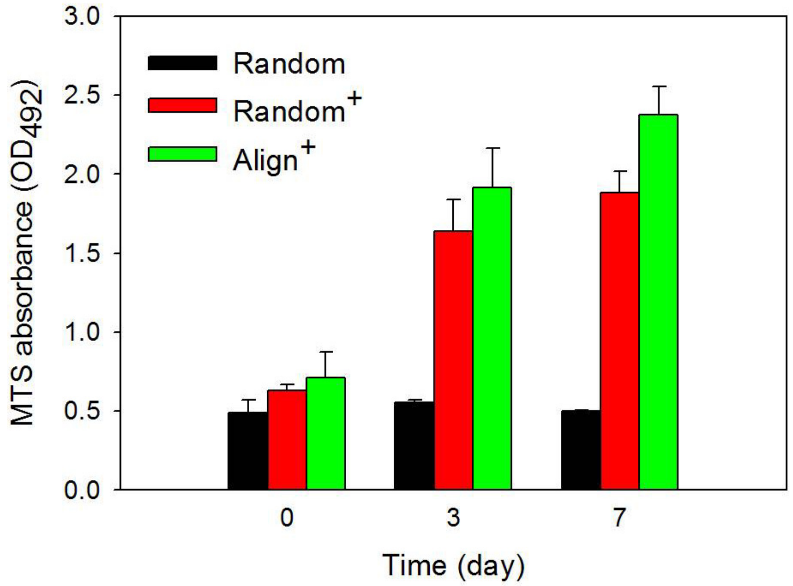

Figure S2. The proliferation of tenocytes on tissue culture polystyrene (TCPS) after cultured with 3-day extract of Random, Random$^+$ or Align$^+$ core-sheath nanofiber membrane scaffold (CSNMS). The viable cell number was determined by MTS assay from solution absorbance at 492 nm (OD_{492}).
